# Genetic determinism of boar taint and relationship with growth traits, meat quality and lesions

**DOI:** 10.1017/S1751731120000105

**Published:** 2020-07

**Authors:** C. Dugué, A. Prunier, M. J. Mercat, M. Monziols, B. Blanchet, C. Larzul

**Affiliations:** 1 GenPhySE, université de Toulouse, INRAE, ENVT, 24, chemin de Borde-Rouge - Auzeville Tolosane 31326, F-31326 Castanet-Tolosan, France; 2 PEGASE, INRAE, Agrocampus Ouest, 16 le clos, F-35590 St-Gilles, France; 3 IFIP, La Motte au Vicomte, F-35650 Le Rheu, France; 4 UEPR, INRAE, Domaine de la Prise, F-35590 Saint-Gilles, France

**Keywords:** androstenone, oestradiol, heritability, aggressiveness, carcass

## Abstract

Breeding entire males is an alternative to surgical castration to improve their welfare. However, entire males may have a major quality defect called boar taint. Boar taint is partly due to the presence of androstenone in fat. In this study, we estimated the genetic parameters between androstenone and production traits to evaluate the consequences of selection against boar taint for traits of interest. We focused on growth traits, meat quality, lesions, hormone levels and computerised tomography measurements in purebred Piétrain (P) or Piétrain cross Large White (X) entire males. The number of measured animals varied from 670 P and 734 X for hormones concentrations to 553 P and 645 X for computerised tomography measurements. Skin lesions were measured on live pigs shortly after mixing, at the end of the fattening period, and on carcasses. Heritabilities of traits measured by tomography ranged from low to high: femur density (P: 0.34, X: 0.69), loin eye area (P: 0.53, X: 0.88) and loin eye density (P: 0.12, X: 0.18). The mean number of lesions at each stage was lower in purebred pigs than in crossbreds (entering the fattening stage 4.01 in P and 4.68 in X; before slaughter 3.72 in P and 4.22 in X; on carcass 4.50 in P and 4.96 in X). We also observed a decrease in the average number of lesions between the two stages in live pigs. We found high genetic correlations between stages in purebred pigs (0.74 to 0.76) but low correlations (−0.30 to 0.29) in crossbred pigs. Selection aiming to decrease fat androstenone is feasible (*h*^2^ = 0.57 in P and *h*^2^ = 0.71 in X). It would have overall positive effects on meat production and quality traits. Selection aiming to reduce plasma oestradiol would strongly reduce the level of fat androstenone (rg = 0.89 in P and rg = 0.84 in X). Selection against oestradiol is easier and less invasive since it would only require a blood sample rather than a fat biopsy in live animals.

## Implications

Castration of piglets involves animal welfare, economic and environmental issues and should be stopped. The aim of reducing compounds responsible for boar taint via genetic selection is to put a halt to castration of piglets. Understanding the genetic relationship between boar taint compounds and meat quality, growth traits and agonistic behaviour should determine how to select against these boar taint compounds without jeopardising other traits of interest. The results of our research suggest that indirect selection against androstenone by reducing the concentration of oestradiol in the blood should enable easier selection against boar taint without detrimental effect on the studied traits.

## Introduction

Surgical castration of piglets is widely practiced in Europe. The advantages are improved meat quality and easier to raise animals due to reduced aggressive and sexual behaviours (for a review, see Von Borell *et al.*, 2009). However, castration is a painful practice which is highly contested for animal welfare reasons. European pig stakeholders consequently decided to consider ending male piglets’ surgical castration without anaesthesia (Anonymous, 2010). Ending surgical castration would also have positive effects on carcass quality (leaner meat), feed efficiency and hence on production costs and environmental footprint.

Entire males may have a meat quality defect called boar taint. It is very well established that two major compounds are responsible for tainted carcasses (For reviews, see Lundström *et al.*, 2009; Parois *et al.*, 2018). Boar taint is mainly due to the accumulation of androstenone and indole compounds, especially skatole, in fat tissues. The positive relationship between androstenone and skatole contents has been largely documented (for a review, see Zamaratskaia and Suires, 2009). Recent observations suggest that plasma oestradiol measured before slaughter would be a good indicator of the stage of sexual maturation and androstenone accumulation in fat (Prunier, *et al.*, 2016).

Numerous environmental factors can influence skatole levels (Wesoly and Weiler, 2012; Parois *et al.*, 2018), whereas genetic factors are particularly important for androstenone. Genetic selection is thus a valuable option to reduce the risk of boar taint. High genetic correlations between fat androstenone and plasma oestradiol have already been estimated (Grindflek *et al.*, 2011; Parois *et al.*, 2015). As plasma oestradiol is easier, cheaper and faster to measure in live animals than fat androstenone, it would be an efficient criterion for selection against boar taint. This selection could also have a positive impact on the behaviour of male pigs by limiting sexual behaviours and aggressiveness, as already suggested in Parois *et al.* (2015). Previous studies estimated low genetic correlations between fat androstenone or skatole level and growth rate or carcass composition (Windig *et al.*, 2012; Strathe *et al.*, 2013). Merks *et al.* (2010) estimated low genetic correlations between fat androstenone and daily gain (0.19) and between fat skatole and daily gain (−0.33). However, up to now, few authors have focused on genetic parameters linking boar taint risk and behavioural and meat quality traits.

Before selecting against fat androstenone or plasma oestradiol in a purebred line, it would also be of interest to evaluate possible consequences in crossbred animals since in pigs, selection is carried out in purebred lines. In addition to boar taint, the evaluation should include phenotypes linked to behaviour, growth and carcass quality.

The aims of the present study were to estimate (1) the genetic parameters concerning androstenone and other traits of interest in a population of pure Piétrain and a population of Piétrain × Large White cross pigs and (2) the consequences of selection against fat androstenone or plasma oestradiol. The results of the present study are based on a data set that was partly used in previous papers (Parois *et al.*, 2015; Parois *et al.*, 2017).

Preliminary results are presented in Dugué *et al.* (2018).

## Material and methods

### Animals and management

Piétrain (P) and Piétrain × Large White (X) entire male pigs were collected on selection and multiplication farms. Both purebred and crossbred pigs were offspring from the same 96 purebred Piétrain sires. Piglets were transported to a central testing station (Le Rheu, France) at 28 to 35 days of age. Entire males were first housed in groups of 6 up to 28 kg on average. At the fattening stage, animals of the same genotypes from two different pens were mixed with free access to water and a space allowance of 1 to 1.2 m^2^/pig. Animals were fed *ad libitum* at an electronic single-space feeder (ACEMA64), with pellets of standard composition (NE =9.5 MJ/kg, total nitrogenous matter = 163 g/kg, digestible lysine content = 0.94 g/MJ NE and digestible tryptophan content = 1.7 g/kg). A total of 670 purebred Piétrain boars and 736 crossbred Piétrain × Large White boars were raised under the same conditions from post weaning to slaughter planned at the target live weight of 112 kg. Before slaughter, the animals were kept in their pen without access to food from the previous day at 1800 h until departure. During transport and lairage at the slaughterhouse, animals of the same genotypes from different pens were mixed. The mean duration between departure from the farm and slaughter was around 170 min with 20 min of transport and 150 min of lairage at the slaughterhouse. Animals were slaughtered in a commercial slaughterhouse, in compliance with national French regulations, by electrical stunning and immediate exsanguination. Carcasses were kept in a cold room at 4°C for 24 h.

### Traits and measurements

Animals were weighed at the beginning of the growing period, around 35 kg of weight and the day before slaughter. Average daily gain (**ADG**) was calculated between 35 and 112 kg. The feed conversion ratio (**FCR**) and average daily feed intake (**ADFI**) were calculated from the total feed consumption recorded by the electronic feeder between 35 and 112 kg. Around 1 week before slaughter, in the morning, a 7 to 9 ml blood sample was collected in EDTA tubes by direct puncture of the external jugular vein. Blood samples were immediately centrifuged at 2500 **g** for 10 min at +4°C, and the plasma was stored at −20°C until analysis of testosterone and 17*β*-oestradiol with RIA kits (testosterone: Immunotech, Prague, Czech Republic, oestradiol: Orion Diagnostica, Espoo, Finland). The detection limit for testosterone was 0.2 ng/ml. The detection limit for oestradiol was 2.5 pg/ml.

On the day of slaughter, after cooling, the carcass was weighed. Carcass yield (**CY**) was calculated as the ratio of cold carcass weight to slaughter weight. The right half-carcasses were then cut following the standard procedure described by Métayer et Daumas (1998). Lean percentage (**L%**) was calculated from the weight of the primal cut using the equation provided by Daumas (2008). The left half-carcasses were analysed by IFIP computerised tomography (**CT**) scanner (Siemens emotion duo, Erlangen Germany) with the following acquisition protocol: 130 kV radiogenic tube voltage, radiogenic tube intensity 30 mA, cutting thickness 3 mm, FOV 500 mm × 500 mm, acquisition matrix 512 × 512, soft tissue reconstruction filter B30s. Tomographic images were analysed with ImageJ software (http://imagej.nih.gov/ij/) which enabled estimation of the lean percentage (**L%CT**), loin eye area (**LEA**), loin eye density (**LED**), femur density (**FemD**) and the ratio of ham muscle volume to femur length (**HFR**).

On the day after slaughter, a piece of backfat was sampled on the carcass in the neck region (between cervical and first dorsal ribs), vacuum-packed and stored at −20°C for further analysis. After thawing, a piece of fat weighing 10 to 20 g was heated and centrifugated at 11 200×**g** for 20 min at +4°C, and a 2 ml sample of the supernatant was used to extract androstenone with methanol. The compound was measured by HPLC (Batorek *et al.*, 2012). Concentrations are expressed per gram of the lipid fraction from adipose tissue. The limit of detection of the method was 0.24, 0.03 and 0.03 μg/g, respectively, for androstenone, skatole and indole (Batorek *et al.*, 2012).

pH was measured with a Xerolyt electrode (Mettler-Toledo, Australia) and a Sydel pH meter (Sydel, France) at 24 h (pH) in the *longissimus dorsi* (Ld) and the *semimembranosus* (Ham). A loin sample (weighing about 130 g) was cut from the right-half carcass at the 13th lumbar vertebra, trimmed, weighed, placed in a polystyrene punnet and stored at 4°C for 48 h at an angle of 40°C. The cut was then removed from the punnet, gently wiped and weighed. Drip loss (**DL**) was calculated as the ratio of weight loss to initial cut weight. The same cut was then stored in the punnet at −20°C for analysis of intramuscular fat (**IF**). The samples were thawed and kept at 5°C until IF was estimated by magnetic resonance imaging (AVANTO 1.5T, Siemens, Erlangen, DE) according to Davenel *et al.* (2012).

Skin lesions were observed at three periods. On live piglets, lesions were measured 48 h after entering the fattening pen (**LESFE**) and on the day before the departure of the first pen mate to the slaughterhouse. Skin lesions were also measured on both sides of the carcass after carcass cooling. Lesions more than 2 cm in length were recorded on both sides. If several small lesions were separated by less than 2 cm, only one lesion was counted.

### Statistical analyses and genetic parameter estimation

Table 1 shows the number of animals, means, SD and minimum and maximum for all traits for purebred and crossbred pigs.

**Table 1:** Number of animals, means and SD for all traits for purebred and crossbred pigs

	Purebred			Crossbred		
Traits	Number	Mean	SD	Number	Mean	SD
ADG	654	939	98.5	717	1038	92.8
FCR	631	2.29	0.15	710	2.25	0.149
ADFI	631	2162	184	710	2342	224
L%	635	65.2	1.77	714	62.7	1.98
CY	637	80.4	1.31	715	79.3	1.25
pH Ld	657	5.58	0.14	734	5.62	0.16
pH Ham	657	5.68	0.18	734	5.72	0.19
IF	555	1.14	0.24	670	1.21	0.26
DL	631	7.3	2.78	709	4.91	1.98
ANDR	654	8.93	0.6	734	9.06	0.62
TES	670	10.2	0.66	733	10.3	0.64
OES	670	11	0.33	731	11.1	0.43
LESFE	642	4.01	0.85	712	4.68	0.77
LESBS	643	3.72	0.806	736	4.22	0.86
LESC	655	4.5	0.86	731	4.96	0.95
L%CT	553	69.7	2.44	646	67.4	2.44
LEA	556	5839	515	647	5363	492
LED	556	65.7	2.38	647	65.2	2.36
FemD	553	1059	56.3	645	1075	55.3
HFR	556	54 229	3830	647	49 299	3087

ADG = average daily gain; FCR = feed conversion ratio; ADFI = average daily feed intake; L% = lean percentage; CY = carcass yield; pH Ld = pH in *Longissimuss Dorsi*; pH Ham = pH in Ham; IF = intramuscular fat; DL = drip loss; ANDR = log(back fat androstenone level); TES = log(plasma testosterone level); OES = log(plasma oestradiol level); LESFE = log(lesions at fattening stage entrance); LESBS = log(lesions before slaughter); LESC = log(lesions on carcass); L%CT = lean percentage with computerised tomography measure; LEA = loin eye area; LED = loin eye density; FemD = femur density; HFR = ham muscle/bone length ratio.

For testosterone, oestradiol and androstenone measurements, the detection limits of the assays were assigned to pigs whose levels were below these limits. Androstenone and sex hormones and skin lesion counts were normalised by logarithmic transformation before analysis. Heritability and genetic correlations were estimated using VCE6 software (Neumaier and Groeneveld, 1998). Supplementary Material Table S1 lists the statistical models used to estimate genetic parameters. Heritabilities and genetic correlations are considered low from 0.0 to 0.4, moderate from 0.4 to 0.6 and high from 0.6 to 1.

## Results and discussion

Estimates of heritabilities, genetic correlations and SE estimated in purebred and crossbred boars with all studied traits are shown in Supplementary Material (Tables S2 to S5).

### Heritability values

For growth traits, the heritabilities estimated in our populations are within the range reported in the literature, with low to moderate values for ADG (P: 0.36, X: 0.50, Table 2), FCR (P: 0.41, X: 0.33) and ADFI (P: 0.47, X: 0.59). Previous heritabilities reported for these traits were 0.03 < *h* ^2^ < 0.49, 0.12 < *h* ^2^ < 0.58 and 0.13 < *h* ^2^ < 0.62, respectively (Clutter, 2011).

**Table 2:** Heritabilities for purebred and crossbred pigs

	Purebred			Crossbred		
Traits	*h* ^2^	SE min	SE max	*h* ^2^	SE min	SE max
ADG	0.36	0.10	0.15	0.50	0.06	0.12
FCR	0.41	0.07	0.11	0.33	0.07	0.09
ADFI	0.47	0.06	0.12	0.59	0.08	0.10
L%	0.82	0.06	0.11	0.81	0.06	0.10
CY	0.34	0.03	0.10	0.49	0.08	0.10
pH Ld	0.43	0.07	0.12	0.26	0.06	0.11
pH Ham	0.34	0.06	0.10	0.28	0.07	0.09
IF	0.44	0.04	0.11	0.44	0.08	0.10
DL	0.62	0.07	0.10	0.26	0.06	0.08
ANDR	0.57	0.04	0.10	0.71	0.04	0.10
TES	0.11	0.03	0.09	0.29	0.04	0.10
OES	0.23	0.07	0.10	0.18	0.07	0.09
LESFE	0.25	0.05	0.10	0.25	0.06	0.10
LESBS	0.14	0.07	0.09	0.20	0.06	0.09
LESC	0.30	0.08	0.11	0.36	0.08	0.10
L%CT	0.75	0.08	0.10	0.71	0.08	0.10
LEA	0.53	0.07	0.11	0.88	0.08	0.11
LED	0.12	0.04	0.08	0.18	0.07	0.10
FemD	0.34	0.10	0.14	0.69	0.08	0.10
HFR	0.67	0.07	0.11	0.76	0.07	0.10

*h* ^2^ = average of the heritabilities estimated for this trait; SE min = minimum standard error estimated for this trait; SE max = maximum standard error estimated for this trait; ADG = average daily gain; FCR = feed conversion ratio; ADFI = average daily feed intake; L% = lean percentage; CY = carcass yield; pH Ld = pH in *Longissimuss Dorsi*; pH Ham = pH in Ham; IF = intramuscular fat; DL = drip loss; ANDR = log(back fat androstenone level); TES = log(plasma testosterone level); OES = log(plasma oestradiol level); LESFE = log(lesions at Fattening stage Entrance); LESBS = log(lesions before slaughter); LESC = log(lesions on carcass); L%CT = lean percentage with computerised tomography measure; LEA = loin eye area; LED = loin eye density; FemD = femur density; HFR = ham muscle/bone length ratio.

The heritabilities for carcass composition, measured either by carcass cut or by CT, were high, between 0.71 and 0.80 for L% and L%CT, compared with values reported in the literature, around 0.5 (Ciobanu *et al.*, 2011). Given the numerous methods used to estimate lean percentage reported in different studies, the range of heritability values for this trait is expected to be wide. The high values found in the present populations can be partly explained by the methodology based either on standardised carcass cuts or on tomography. The range of heritability values could also be attributed to the segregation of the n allele of the RYR1 gene present in the French Piétrain pig population, which significantly affects lean meat content (Salmi *et al.*, 2010). Computerised tomography was also used to provide different measurements, such as LEA, LED, FemD and HFR. The LEA had a moderate to high heritability (P: 0.53, X: 0.88) which tended to be higher than the heritability reported in the literature (0.47 < *h* ^2^ < 0.48, Ciobanu *et al.*, 2011). For lean meat content, CT scanning provides a more accurate measurement of muscle area, at least compared to muscle thickness measured by ultrasound. Heritability was low for LED (P: 0.12, X: 0.18). In lamb, Karamichou *et al.* (2006) estimated low to high heritability (0.34 to 0.85) for a similar trait depending on the measurement location. The low heritability value estimated in the present study could be linked to the method of measurement, which was performed on cold carcasses instead of live animals. For FemD, the heritability was low in purebred pigs (*h* ^2^ = 0.38) and high in crossbred pigs (*h* ^2^ = 0.68). In lambs, it was previously shown that the heritability of bone density evaluated by the CT scanner was moderate (0.4 to 0.5, Karamichou *et al.*, 2006). FemD could be related to leg weakness, although this has not been previously demonstrated in pig. Heritability estimates reported in the literature for leg weakness scores are of the same magnitude. For example, Huang *et al.* (1995) reported heritabilities of 0.37 to 0.60 for a leg weakness score in Duroc, Yorkshire and Landrace pigs.

The heritability for ham/femur length ratio was high in both purebred and crossbred pigs. Ham muscle/bone length ratio measures ham conformation, and, like carcass lean meat content, it is influenced by the RYR1 n allele, with high development of lean tissues in the leg (Fisher *et al.*, 2000), which could partially explain the heritability estimate.

Heritabilities estimated for meat quality traits tended to be within the range of values reported in the literature for pig meat (Sosnicki, 2016). For pH, we estimated that the heritability in the present populations ranged from 0.26 to 0.43 in ham and *longissimus dorsi.* For IF measured by MRI, we estimated a heritability of 0.44 in purebred and crossbred pigs. These estimates are in good agreement with previous published values (0.10 < *h* ^2^ < 0.40 for pH and 0.25 < *h* ^2^ < 0.85 for IF, Sosnicki, 2016).

Our data reveal low to moderate heritability of the number of skin lesions measured on live pigs shortly after mixing (0.25 in both P and X pigs, Table 2) or on carcasses (P: 0.30, X: 0.36). This is in good agreement with data published by Turner *et al.* (2006), who estimated a heritability of 0.22 in pure Large White and Landrace females and entire male pigs. Focusing on the lesions located in the anterior part of the body, the heritability estimate was 0.11 in one study (Turner *et al.*, 2006), 0.26 in a second (Turner *et al.*, 2009), 0.08 in a third (Desire *et al.*, 2015) and 0.13 in a fourth study (Desire *et al.*, 2016). The animals in the four studies were observed in a situation similar to that in our study, shortly after mixing at about 2 to 3 months of age. After several weeks of social stability, the heritability measured in the present study was low (P: 0.14, X: 0.20) comparable to that observed by Desire *et al.* (2015) for anterior skin lesions measured 5 weeks after grouping (0.18) but lower than that observed by Turner *et al.* (2009) also for anterior lesions but 3 weeks after grouping (0.43).

Androstenone has moderate heritability in purebred pigs (*h* ^2^ = 0.57) and high heritability in crossbred pigs (*h* ^2^ = 0.71). It is well known that androstenone is genetically determined and the heritability estimates reported in the literature are moderate to high (for a review, see Parois *et al.* 2018). Testosterone and oestradiol levels had low heritability in both genetic types (between 0.1 and 0.3) in agreement with Grindflek *et al.* (2011).

We looked at the genetic correlation between purebred and crossbred (*r*_pc_). In our study, the average *r*_pc_ is 0.77.

In a review (Wientjes and Calus, 2017), the average of the *r*_pc_ for studies in which the environment and the methods of measurement are identical for purebred and crossbred, the average *r*_pc_ is 0.66. We therefore have a *r*_pc_ better than average *r*_pc_, which can be explained by the structure of our population with only two breeds.

### Genetic correlations with computerised tomography traits

L% was highly correlated with L%CT (P: 0.91, X: 0.83), and, in general, for these two measurement methods, correlations with the other studied traits were close (see Supplementary Material Table S2), which can be explained by the fact that the two measurement methods produced highly correlated results even though mean values differed slightly (69.7 *v*. 65.2 in P pigs, 67.4 *v*. 62.7 in X pigs, Table 1).

As expected, both LEA and HFR had high positive genetic correlations with L% and L%CT (Table 3) because they are related to muscle development, either in the loin part or in the leg part. Given the genetic parameters, it would be quite difficult to differentially select the back and the ham for different conformations, putting more emphasis on either the back or the ham.

**Table 3:** Genetic correlations with CT traits for purebred (P) and crossbred pigs (X)

	LEA P		LED P		FemD P		HFR P	
Traits	rg	SE	rg	SE	rg	SE	rg	SE
ADG P	−0.09	0.23	0.17	0.25	0.45	0.18	−0.02	0.16
ADG X	0.19	0.23	0.87	0.08	−0.09	0.30	0.10	0.16
FCR P	−0.58	0.10	0.76	0.23	0.44	0.22	−0.72	0.11
FCR X	−0.56	0.13	0.63	0.33	0.83	0.11	−0.77	0.21
ADFI P	−0.53	0.11	0.39	0.50	0.72	0.15	−0.58	0.13
ADFI X	−0.32	0.16	0.62	0.44	0.72	0.19	−0.52	0.21
L% P	0.62	0.10	−0.59	0.40	−0.59	0.18	0.72	0.08
L% X	0.50	0.15	−0.59	0.36	−0.68	0.20	0.69	0.12
CY P	0.35	0.14	−0.85	0.29	−0.15	0.20	0.26	0.14
CY X	0.44	0.19	−0.50	0.56	−0.04	0.24	0.39	0.19
pH Ld P	0.13	0.18	−0.29	0.33	0.30	0.22	−0.31	0.10
pH Ld X	−0.16	0.23	−0.56	0.32	0.41	0.33	−0.31	0.11
pH Ham P	0.17	0.12	−0.84	0.28	0.36	0.24	−0.02	0.15
pH Ham X	−0.09	0.22	−0.57	0.34	0.62	0.30	−0.25	0.13
IF P	−0.38	0.15	0.06	0.31	0.33	0.17	−0.54	0.11
IF X	−0.11	0.24	−0.35	0.31	0.32	0.18	−0.58	0.15
DL P	0.09	0.14	−0.28	0.26	−0.24	0.18	0.36	0.13
DL X	0.15	0.19	0.01	0.43	−0.42	0.17	0.25	0.16
ANDR P	−0.31	0.15	0.39	0.36	0.18	0.18	−0.51	0.09
ANDR X	−0.10	0.17	−0.11	0.46	−0.34	0.24	−0.10	0.13
TES P	−0.45	0.33	−0.96	0.29	−0.30	0.33	−0.09	0.24
TES X	−0.48	0.24	−0.74	0.41	−0.63	0.16	−0.19	0.22
OES P	−0.46	0.21	0.42	0.31	0.00	0.27	−0.38	0.08
OES X	−0.60	0.24	0.31	0.34	0.02	0.42	−0.32	0.12
LESFE P	0.25	0.22	0.42	0.32	−0.11	0.27	0.06	0.09
LESFE X	0.11	0.20	−0.38	0.48	−0.59	0.30	0.21	0.20
LESBS P	−0.05	0.14	−0.09	0.33	0.41	0.36	−0.24	0.26
LESBS X	0.36	0.23	−0.83	0.28	0.03	0.35	0.33	0.21
LESC P	0.03	0.20	−0.48	0.30	0.08	0.24	−0.10	0.18
LESC X	0.19	0.25	−0.12	0.29	0.26	0.28	−0.17	0.24
L%CT P	0.55	0.11	−0.56	0.12	−0.79	0.16	0.79	0.07
L%CT X	0.60	0.14	−0.63	0.37	−0.87	0.16	0.70	0.16
LEA P			0.04	0.23	−0.01	0.19	0.67	0.09
LEA X			0.28	0.34	−0.15	0.15	0.56	0.22
LED P					0.64	0.51	−0.46	0.27
LED X					0.55	0.20	0.03	0.27
FemD P							−0.55	0.20
FemD X							−0.46	0.25

CT = computerised tomography; LEA = loin eye area; LED = loin eye density; FemD = femur density; HFR = ham muscle/bone length ratio; rg = genetic correlation; SE = standard error; ADG = average daily gain; FCR = feed conversion ratio; ADFI = average daily feed intake; L% = lean percentage; CY = carcass yield; pH Ld = pH in *Longissimuss Dorsi*; pH Ham = pH in Ham; IF = intramuscular fat; DL = drip loss; ANDR = log(back fat androstenone level); TES = log(plasma testosterone level); OES = log(plasma oestradiol level); LESFE = log(lesions at Fattening stage Entrance); LESBS = log(lesions before slaughter); LESC = log(lesions on carcass); L%CT = lean percentage with computerised tomography measure.

Loin eye density and femur density were generally positively correlated with growth traits, with low (rg = 0.17 between LED and ADG in P pigs) to high rg (rg = 0.76 between LED and FCR and rg = 0.72 between FemD and ADFI in P pigs, rg = 0.87 between LED and ADG and rg = 0.83 between FemD and FCR in X pigs). The relationship with bone characteristics and growth traits or lean meat content was poorly described. In their study on lambs, Karamichou *et al.* (2006) did not report genetic correlations between bone density measured by CT scanning and growth traits, but in the line selected for higher lean content, bone density was lower than in the line selected for higher fat content. The present findings confirm that selection for better feed efficiency or lean meat content would have detrimental effects on bone density and hence on leg soundness since the genetic correlations were highly positive with FCR (P: 0.44, X: 0.83) or highly negative with lean meat content as estimated by L% (P: −0.59, X: −0.68) or L%CT (P: −0.79, X: −0.87).

Karamichou *et al.* (2006) reported a high negative genetic correlation between muscle density and IF in lamb, suggesting that it would be possible to select on meat quality traits using the results of CT scans. The genetic correlations estimated between LED and pH were negative from low to high (−0.29 between LED and pH ld in P and rg = −0.84 in X between LED and pH Ham). Further investigation will be required to understand if this variability is only due to genetic type. Conversely, muscle density measured on live lambs was positively correlated with meat pH (Karamichou *et al.*, 2006). The lack of consistency between results obtained with purebred and crossbred pigs and with different muscles is an obstacle to using muscle density as a criterion for selecting based on meat quality traits.

### Genetic correlation with for skin lesions

In the present study, the number of lesions was measured at three stages: at the beginning of the fattening stage, before slaughter and on carcasses. To our best of our knowledge, this is the first study to show such results.

The mean number of lesions at each stage was lower in purebred pigs than in crossbred pigs (Table 1), which could be explained by lower aggressiveness in purebred pigs. Due to their sensitivity to stress when the n allele of the RYR1 gene is present on both chromosomes, Pietrain pigs may have been indirectly selected for lower aggressiveness. Indeed, intense fighting could have been fatal for such pigs.

Before slaughter, we expected an increase in the number of lesions, as the animals were sexually mature. On the contrary, we observed lower averages (Table 1). This can be explained by the fact that the first measurement was made shortly after grouping unacquainted pigs from two pens, whereas the second measurement was made using groups of pigs formed several weeks previously. Indeed, grouping unacquainted pigs is known to trigger aggression and consequently skin lesions, whereas the number of aggressions and skin lesions decreases over time in stable groups (Turner *et al.*, 2006; Prunier *et al.*, 2013; Tallet *et al.*, 2013). The number of lesions on the carcasses was relatively high, in good agreement with the fact that pigs were grouped with unacquainted pigs in the lairage area.

We estimated high genetic correlations between periods (0.74 < rg < 0.76, Table 4) in purebred pigs but low correlations between periods (−0.30 < rg < 0.29) in crossbred pigs. A study by Turner *et al.* (2009) reported a low genetic correlation (0.28) between anterior skin lesions measured shortly after mixing and 3 weeks later, whereas the study by Desire *et al.* (2015) reported a high correlation (0.76). Animals in the study by Turner *et al.* (2009) were intact or castrated males and females from purebred Yorkshire and crossbred Yorkshire × Landrace genotypes, whereas pigs in the study by Desire *et al.* (2015) were females and castrates from a PIC commercial herd. The reasons for such differences between studies may be due to differences not only in sexual type or in genotype but also in the housing environment.

**Table 4:** Genetic correlations with lesion scores for purebred (P) and crossbred pigs (X)

	LESFE P		LESBS P		LESC P	
Traits	rg	SE	rg	SE	rg	SE
ADG P	−0.47	0.25	−0.49	0.52	−0.20	0.22
ADG X	−0.53	0.32	−0.19	0.44	−0.73	0.18
FCR P	0.12	0.22	0.54	0.36	0.22	0.21
FCR X	0.19	0.42	0.40	0.54	0.47	0.22
ADFI P	−0.55	0.22	−0.08	0.32	0.00	0.20
ADFI X	−0.40	0.35	0.21	0.46	−0.31	0.20
L% P	0.42	0.20	−0.32	0.19	0.13	0.17
L% X	0.20	0.28	−0.50	0.29	0.29	0.20
CY P	0.24	0.21	−0.16	0.24	0.20	0.22
CY X	−0.03	0.23	0.06	0.26	0.31	0.22
pH Ld P	0.50	0.21	0.60	0.30	0.16	0.20
pH Ld X	0.57	0.28	0.34	0.36	0.62	0.20
pH Ham P	0.26	0.17	0.39	0.28	0.15	0.19
pH Ham X	0.23	0.21	0.24	0.27	0.64	0.16
IF P	−0.25	0.26	−0.21	0.30	−0.01	0.19
IF X	0.11	0.25	−0.21	0.30	0.40	0.21
DL P	−0.22	0.18	−0.11	0.11	−0.05	0.09
DL X	−0.01	0.19	−0.22	0.31	0.15	0.17
ANDR P	0.24	0.09	0.21	0.12	0.15	0.19
ANDR X	−0.03	0.32	−0.53	0.12	0.18	0.25
TES P	0.21	0.32	0.82	0.36	0.50	0.42
TES X	0.67	0.29	0.34	0.50	0.39	0.25
OES P	0.21	0.28	0.38	0.39	0.48	0.27
OES X	0.04	0.32	0.20	0.45	0.66	0.22
L%CT P	0.09	0.17	−0.62	0.25	0.02	0.07
L%CT X	0.27	0.32	−0.58	0.39	0.06	0.10
LEA P	0.25	0.22	−0.05	0.14	0.03	0.20
LEA X	0.00	0.38	0.18	0.18	−0.01	0.21
LED P	0.42	0.32	−0.09	0.33	−0.48	0.30
LED X	0.40	0.38	0.89	0.10	−0.69	0.21
FemD P	−0.11	0.27	0.41	0.36	0.08	0.24
FemD X	−0.01	0.27	0.02	0.40	−0.02	0.20
HFR P	0.06	0.09	−0.24	0.26	−0.10	0.18
HFR X	0.47	0.29	−0.17	0.38	−0.21	0.23
LESFE P			0.74	0.16	0.74	0.28
LESFE X			−0.30	0.30	0.23	0.30
LESBS P					0.76	0.25
LESBS X					0.29	0.32

LESFE = log(lesions at fattening stage entrance); LESBS = log(lesions before slaughter); LESC = log(lesions on carcass); rg = genetic correlation; SE = standard error; ADG = average daily gain; FCR = feed conversion ratio; ADFI = average daily feed intake; L% = lean percentage; CY = carcass yield; pH Ld = pH in *Longissimuss Dorsi*; pH Ham = pH in Ham; IF = intramuscular fat; DL = drip loss; ANDR = log(back fat androstenone level); TES = log(plasma testosterone level); OES = log(plasma oestradiol level); L%CT = lean percentage with computerised tomography measure; LEA = loin eye area; LED = loin eye density; FemD = femur density; HFR = ham muscle/bone length ratio.

The genetic correlation between LESFE and ADG was negative and moderate in both types of pigs (P: −0.47, X: −0.53) similar to that in a previous experiment (−0.34) 24 h after mixing in Large White and Landrace pigs (Turner *et al.*, 2006) but more marked than in a more recent one (0.12) by Desire *et al.* (2015). However, Desire *et al.* (2015) distinguished between the location of the lesions on the body and 0.12 was for the front part, and in addition, they calculated the growth rate for the whole lifetime. In the present study, the correlation between skin lesions at the end of the fattening period and ADG estimated was similar in P (−0.49) but less marked in X (−0.19) pigs. Conversely, a positive correlation (0.31) between anterior skin lesions and lifespan was estimated by Desire *et al.* (2015). Again, a difference in the environmental conditions including the feeding system may explain the discrepancy between the two studies. Indeed, in our study, pigs had free access to an electronic single-space feeder that protected them when they were feeding and with a relatively low occupation rate (10 to 12 animals per feeder) so that competition for feeding was probably low, as already pointed out by Parois *et al.* (2017). In this situation, it is likely that being aggressive has no advantage. In the study by Desire *et al.* (2015), the pigs were fed *ad libitum* but the number of feeders available per pig was not mentioned. However, it did mention that the space allowance was 0.65 m^2^/pig, which is low and may limit access to the feeder and hence increase competition between pigs. The present analysis of the genetic correlations between skin lesions and feed intake produced similar results to the situation between skin lesions and the growth rate, which is to be expected since growth rate depends on feed intake.

The correlations between pH and lesions on carcasses were high in crossbred pigs (0.62 and 0.64, respectively, for Ld and Ham) and surprisingly low for purebred pigs (0.16 and 0.15, respectively, for Ld and Ham). A positive correlation was expected since when animals fight, they consume glycogen and the meat pH tends to increase. The low correlation in purebred pigs might be explained by the fact that Piétrain pigs are sensitive to stress and struggle less than the others.

### Genetic correlations with androstenone and oestradiol

The genetic correlations between fat androstenone and plasma oestradiol were very high (P: 0.89, X: 0.96, Table 5). The genetic correlations between fat androstenone and plasma testosterone ranged from low to high (P: 0.80, X: 0.27). Except one, our results are in agreement with those reported by Grindflek *et al.* (2011), who estimated high genetic correlations between fat androstenone and plasma oestradiol in purebred animals (0.90 in Landrace and 0.83 in Duroc) and between fat androstenone and plasma testosterone (0.95 in Landrace and 0.80 in Duroc). Taking the high genetic correlations between fat androstenone and plasma oestradiol into account, both traits could be used to select against boar taint. Therefore, considering the genetic correlations between traits, we extrapolated the expected effects of selection aimed at reducing fat androstenone or plasma oestradiol on production and meat quality traits in purebred pigs and crossbred pigs.

**Table 5:** Genetic correlations with androstenone and oestradiol for purebred (P) and crossbred pigs (X)

	ANDR P		OES P	
Traits	rg	SE	rg	SE
ADG P	−0.16	0.23	−0.18	0.37
ADG X	0.16	0.24	0.01	0.33
FCR P	0.47	0.15	0.55	0.21
FCR X	0.51	0.16	0.49	0.28
ADFI P	0.20	0.08	0.34	0.17
ADFI X	0.39	0.12	0.33	0.09
L% P	−0.26	0.08	−0.18	0.12
L% X	−0.37	0.14	−0.38	0.22
CY P	−0.15	0.17	0.10	0.10
CY X	−0.49	0.20	−0.46	0.17
pH Ld P	−0.20	0.14	−0.03	0.25
pH Ld X	−0.10	0.24	0.32	0.31
pH Ham P	−0.40	0.16	−0.48	0.20
pH Ham X	−0.23	0.23	−0.12	0.32
IF P	−0.04	0.17	−0.16	0.23
IF X	0.32	0.19	0.07	0.30
DL P	0.08	0.10	0.02	0.17
DL X	0.40	0.21	0.17	0.30
TES P	0.80	0.23	0.89	0.09
TES X	0.40	0.24	0.80	0.21
OES P	0.89	0.09		
OES X	0.84	0.18		
LESFE P	0.24	0.09	0.21	0.28
LESFE X	0.89	0.10	0.99	0.13
LESBS P	0.21	0.12	0.38	0.39
LESBS X	0.10	0.26	−0.21	0.27
LESC P	0.15	0.19	0.48	0.27
LESC X	−0.26	0.25	−0.25	0.27
L%CT P	−0.37	0.07	−0.36	0.18
L%CT X	−0.47	0.12	−0.44	0.16
LEA P	−0.31	0.15	−0.46	0.21
LEA X	−0.50	0.17	−0.18	0.27
LED P	0.39	0.36	0.42	0.31
LED X	0.14	0.19	−0.24	0.36
FemD P	0.18	0.18	0.00	0.27
FemD X	0.27	0.17	−0.16	0.23
HFR P	−0.51	0.09	−0.38	0.08
HFR X	−0.34	0.15	−0.12	0.18

ANDR = log(back fat androstenone level); OES = log(plasma oestradiol level); rg = genetic correlation; SE = standard error; ADG = average daily gain; FCR = feed conversion ratio; ADFI = average daily feed intake; L% = lean percentage; CY = carcass yield; pH Ld = pH in *Longissimuss Dorsi*; pH Ham = pH in Ham; IF = intramuscular fat; DL = drip loss; TES = log(plasma testosterone level); LESFE = log(lesions at fattening stage entrance); LESBS = log(lesions before slaughter); LESC = log(lesions on carcass); L%CT = lean percentage with computerised tomography measure; LEA = loin eye area; LED = loin eye density; FemD = femur density; HFR = ham muscle/bone length ratio.

Selection to reduce fat androstenone in purebred pigs would have favourable effects on the ham muscle/bone length ratio (rg = −0.51), FCR (rg = 0.47) and pH in ham (rg = −0.40) and unfavourable effects on testosterone concentration (rg = 0.80) in purebred pigs.

Selection against back fat androstenone in crossbred pigs would have favourable effects on the number of skin lesions shortly after mixing (rg = 0.89), on the FCR (rg = 0.51), CY (rg = −0.49), LEA (rg = −0.50), DL (rg = 0.40), ADFI (rg = 0.39) and lean percentage (L%CT rg = −0.47, L% rg = −0.37). It would have unfavourable effects on plasma testosterone (rg = 0.40).

Selection to reduce plasma oestradiol in purebred animals would have favourable effects on fat androstenone (rg = 0.89), FCR (rg = 0.55), LEA (rg = −0.46), pH in Ham (rg = −0.48) and on skin lesion number on carcasses (rg = 0.48). It would have unfavourable effects on plasma testosterone (rg = 0.82).

Selection against oestradiol in crossbred pigs would have favourable effects on fat androstenone (rg = 0.80), on the FCR (rg = 0.49), L% CT (rg = −0.44), carcass yield (rg = −0.46) and the number of skin lesions shortly after mixing (rg = 0.99) and unfavourable effect on testosterone concentration (rg = 0.57).

To the best of our knowledge, these results based on genetic correlations between these traits are the first ones in the existing literature.

## Conclusion

Estimating the genetic parameters for a large number of production traits (carcass composition, behaviour-related, sex hormones and meat quality) provided new insights that will be useful in breeding entire males. We confirmed that selection against the risk of boar taint, focusing on androstenone, would not be detrimental to production traits or carcass composition in either purebred or crossbred animals. Some meat quality traits such as pH would be improved by such a selection. As entire males are leaner than castrated males, in breeding schemes, more consideration should be given to the organoleptic qualities of meat from entire males beyond boar taint risk. Selection for decreasing plasma oestradiol would be an interesting alternative way to reduce the level of fat androstenone at slaughter as it is technically easier to collect a blood sample from live animals than to take a sample of fat during biopsy. Selection against plasma oestradiol would also have a beneficial effect on the behaviour of entire males with less aggressive behaviours. Accounting for the influence of steroids on skatole storage would also have a beneficial effect on fat skatole. However, selection for reduced androstenone or plasma oestradiol may have an impact on reproduction ability, which thus requires further study.

## Software and data repository resources

None of the data were deposited in an official repository.

## Supplementary material

For supplementary material accompanying this paper visit https://doi.org/10.1017/S1751731120000105. click here to view supplementary material
