# Supplementary material for: Genetic determinism of boar taint and relationship with growth traits, meat quality and lesions
Source: Animal. 2020 Feb 13;14(7):1333–41. doi: 10.1017/S1751731120000105 (PMC7301229; doi:10.1017/S1751731120000105)
Supplement: Supplementary file 1 [file S1751731120000105sup001.docx]

SUPPLEMENTARY INFORMATION

*animal* journal

**Genetic determinism of boar taint and relationship with growth traits, meat quality and lesions**

C. Dugué1, A. Prunier2, M.J. Mercat3, M. Monziols3, B. Blanchet4 and C. Larzul1

1 GenPhySE, université de Toulouse, INRAE, ENVT, F-31326, Castanet-Tolosan, France

2 PEGASE, INRAE, Agrocampus Ouest, St-Gilles, France

3 IFIP, Le Rheu, France

*4* UEPR, INRAE, Le Rheu, France.

Corresponding author: Claire Dugué. Email: claire.dugue@inrae.fr

Table S1 Statistical models used to estimate genetic parameters for purebred and crossbred pigs

|  | Random effects | |  | Fixed effects | | |  | Covariate | |
| --- | --- | --- | --- | --- | --- | --- | --- | --- | --- |
|  |  |  |  |  | Date of | |  | Live weight at | |
|  | Animal | Pen |  | Batch | Blood sampling | Slaughter |  | Test start | End of fattening |
| ADG | x | x |  | x |  |  |  | x |  |
| FCR | x | x |  | x |  |  |  | x |  |
| ADFI | x | x |  | x |  |  |  | x |  |
| L% | x | x |  | x |  |  |  |  | x |
| CY | x | x |  | x |  |  |  |  | x |
| pH Ld | x |  |  |  |  | x |  |  | x |
| pH Ham | x |  |  |  |  | x |  |  | x |
| IF | x |  |  | x |  |  |  |  | x |
| DL | x |  |  |  |  | sx |  |  | x |
| ANDR | x |  |  |  |  | x |  |  | x |
| TES | x |  |  |  | x |  |  |  | x |
| OES | x |  |  |  | x |  |  |  | x |
| LESFE | x | x |  | x |  |  |  |  | x |
| LESBS | x | x |  | x |  |  |  |  | x |
| LESC | x |  |  |  |  | x |  |  | x |
| L%CT | x | x |  | x |  |  |  |  | x |
| LEA | x |  |  |  |  | x |  |  | x |
| LED | x |  |  |  |  | x |  |  | x |
| FemD | x | x |  | x |  |  |  |  | x |
| HFR | x | x |  | x |  |  |  |  | x |

ADG: Average Daily Gain, FCR: Feed Conversion ratio, ADFI: Average Daily Feed Intake, L%: Lean Percentage, CY: Carcass Yield, pH Ld: pH in *Longissimuss Dorsi*, pH Ham: pH in Ham IF: Intramuscular Fat, ANDR: log(Back fat androstenone level), TES: log(plasma testosterone level), OES: log(plasma œstradiol level), LESFE: log(Lesions at Fattening stage Entrance), LESBS: log(Lesions Before Slaughter), LESC: log(Lesions on Carcass), L%CT: Lean percentage with computerized tomography measure, DL: Drip Loss, LEA Loin Eye Area, LED: Loin Eye Density, FemD: Femur Density, HFR: Ham muscle/bone length ratio.

Table S2 Estimates of heritabilities (diagonal), genetic correlations (above diagonal), and SE estimated (below diagonal) in purebred (p) and crossbred (x) boars with growth traits and carcass composition.

|  | Growth traits | | | | | |  | Carcass Composition | | | | |
| --- | --- | --- | --- | --- | --- | --- | --- | --- | --- | --- | --- | --- |
|  | ADG p | ADG x | FCR p | FCR x | ADFI p | ADFI x | L% p | | L% x | CY p | CY x |  |
| ADG p | **0.36** | 0.50 | -0.42 | 0.30 | 0.77 | 0.68 | -0.32 | | -0.28 | 0.39 | 0.34 |  |
| ADG x |  | **0.50** | -0.12 | -0.05 | 0.46 | 0.86 | -0.42 | | -0.46 | 0.23 | -0.03 |  |
| FCR p | 0.07 | 0.26 | **0.41** | -0.12 | 0.52 | 0.38 | -0.58 | | -0.47 | -0.40 | -0.23 |  |
| FCR x | 0.23 | 0.19 |  | **0.33** | 0.76 | 0.49 | -0.60 | | -0.50 | -0.13 | 0.07 |  |
| ADFI p | 0.08 | 0.26 | 0.13 | 0.14 | **0.47** | 0.97 | -0.77 | | -0.69 | 0.03 | -0.02 |  |
| ADFI x | 0.27 | 0.05 | 0.15 | 0.12 |  | **0.59** | -0.76 | | -0.66 | 0.06 | -0.14 |  |
| L% p | 0.16 | 0.15 | 0.12 | 0.16 | 0.07 | 0.12 | **0.82** | | 0.98 | 0.33 | 0.04 |  |
| L% x | 0.20 | 0.11 | 0.19 | 0.14 | 0.13 | 0.08 |  | | **0.81** | 0.39 | 0.25 |  |
| CY p | 0.25 | 0.26 | 0.19 | 0.22 | 0.14 | 0.14 | 0.12 | | 0.09 | **0.34** | 0.93 |  |
| CY x | 0.31 | 0.19 | 0.22 | 0.17 | 0.15 | 0.12 | 0.15 | | 0.11 |  | **0.49** |  |
| pH Ld p | 0.29 | 0.35 | 0.14 | 0.31 | 0.18 | 0.21 | 0.16 | | 0.22 | 0.21 | 0.24 |  |
| pH Ld x | 0.37 | 0.23 | 0.23 | 0.30 | 0.20 | 0.20 | 0.19 | | 0.16 | 0.23 | 0.19 |  |
| pH Ham p | 0.22 | 0.28 | 0.19 | 0.27 | 0.23 | 0.19 | 0.09 | | 0.17 | 0.15 | 0.22 |  |
| pH Ham x | 0.27 | 0.23 | 0.20 | 0.28 | 0.22 | 0.19 | 0.18 | | 0.13 | 0.22 | 0.16 |  |
| IF p | 0.28 | 0.25 | 0.20 | 0.21 | 0.13 | 0.16 | 0.13 | | 0.19 | 0.14 | 0.15 |  |
| IF x | 0.34 | 0.18 | 0.19 | 0.19 | 0.11 | 0.14 | 0.14 | | 0.13 | 0.11 | 0.11 |  |
| DL p | 0.27 | 0.22 | 0.14 | 0.19 | 0.15 | 0.17 | 0.11 | | 0.18 | 0.16 | 0.20 |  |
| DL x | 0.31 | 0.23 | 0.26 | 0.21 | 0.16 | 0.18 | 0.16 | | 0.16 | 0.23 | 0.18 |  |
| ANDR p | 0.23 | 0.24 | 0.15 | 0.16 | 0.08 | 0.12 | 0.08 | | 0.14 | 0.17 | 0.20 |  |
| ANDR x | 0.28 | 0.15 | 0.18 | 0.17 | 0.22 | 0.14 | 0.19 | | 0.12 | 0.22 | 0.14 |  |
| TES p | 0.37 | 0.21 | 0.15 | 0.32 | 0.32 | 0.29 | 0.26 | | 0.34 | 0.29 | 0.30 |  |
| TES x | 0.42 | 0.23 | 0.26 | 0.10 | 0.28 | 0.18 | 0.23 | | 0.15 | 0.17 | 0.18 |  |
| OES p | 0.37 | 0.33 | 0.21 | 0.28 | 0.17 | 0.09 | 0.12 | | 0.22 | 0.10 | 0.17 |  |
| OES x | 0.58 | 0.29 | 0.41 | 0.36 | 0.17 | 0.09 | 0.24 | | 0.15 | 0.25 | 0.17 |  |
| LESFE p | 0.25 | 0.32 | 0.22 | 0.42 | 0.22 | 0.35 | 0.20 | | 0.28 | 0.21 | 0.23 |  |
| LESFE x | 0.22 | 0.13 | 0.27 | 0.26 | 0.24 | 0.22 | 0.21 | | 0.18 | 0.22 | 0.17 |  |
| LESBS p | 0.52 | 0.44 | 0.36 | 0.54 | 0.32 | 0.46 | 0.19 | | 0.29 | 0.24 | 0.26 |  |
| LESBS x | 0.20 | 0.19 | 0.27 | 0.22 | 0.28 | 0.24 | 0.23 | | 0.18 | 0.21 | 0.20 |  |
| LESC p | 0.22 | 0.18 | 0.21 | 0.22 | 0.20 | 0.20 | 0.17 | | 0.20 | 0.22 | 0.22 |  |
| LESC x | 0.35 | 0.21 | 0.20 | 0.22 | 0.26 | 0.15 | 0.24 | | 0.22 | 0.24 | 0.26 |  |
| L%CT p | 0.18 | 0.13 | 0.03 | 0.09 | 0.07 | 0.17 | 0.03 | | 0.09 | 0.20 | 0.19 |  |
| L%CT x | 0.19 | 0.13 | 0.21 | 0.18 | 0.09 | 0.12 | 0.06 | | 0.05 | 0.22 | 0.17 |  |
| LEA p | 0.23 | 0.23 | 0.10 | 0.13 | 0.11 | 0.16 | 0.10 | | 0.15 | 0.14 | 0.19 |  |
| LEA x | 0.24 | 0.15 | 0.17 | 0.19 | 0.16 | 0.13 | 0.12 | | 0.11 | 0.12 | 0.09 |  |
| LED p | 0.25 | 0.08 | 0.23 | 0.33 | 0.50 | 0.44 | 0.40 | | 0.36 | 0.29 | 0.56 |  |
| LED x | 0.25 | 0.30 | 0.33 | 0.29 | 0.47 | 0.34 | 0.23 | | 0.21 | 0.24 | 0.22 |  |
| FemD p | 0.18 | 0.30 | 0.22 | 0.11 | 0.15 | 0.19 | 0.18 | | 0.20 | 0.20 | 0.24 |  |
| FemD x | 0.24 | 0.15 | 0.21 | 0.17 | 0.20 | 0.22 | 0.14 | | 0.11 | 0.16 | 0.15 |  |
| HFR p | 0.16 | 0.16 | 0.11 | 0.21 | 0.13 | 0.21 | 0.08 | | 0.12 | 0.14 | 0.19 |  |
| HFR x | 0.13 | 0.09 | 0.24 | 0.22 | 0.13 | 0.16 | 0.14 | | 0.09 | 0.18 | 0.10 |  |

ADG: Average Daily Gain, FCR: Feed Conversion ratio, ADFI: Average Daily Feed Intake, L%: Lean Percentage, CY: Carcass Yield, pH Ld: pH in *Longissimuss Dorsi*, pH Ham: pH in Ham IF: Intramuscular Fat, ANDR: log(Back fat androstenone level), TES: log(plasma testosterone level), OES: log(plasma œstradiol level), LESFE: log(Lesions at Fattening stage Entrance), LESBS: log(Lesions Before Slaughter), LESC: log(Lesions on Carcass), L%CT: Lean percentage with computerized tomography measure, DL: Drip Loss, LEA Loin Eye Area, LED: Loin Eye Density, FemD: Femur Density, HFR: Ham muscle/bone length ratio, SE : standard error.

Table S3 Estimates of heritabilities (diagonal), genetic correlations (above diagonal), and SE estimated (below diagonal) in purebred (p) and crossbred (x) boars with meat quality.

|  | | | | | | Meat quality | | | | | | |
| --- | --- | --- | --- | --- | --- | --- | --- | --- | --- | --- | --- | --- |
|  | pH Ld p | pH Ld x | pH Ham p | pH Ham x | IF p | | IF x | DL p | DL x | ANDR p | ANDR x |  |
| ADG p | -0.13 | 0.38 | -0.08 | 0.56 | 0.27 | | 0.34 | -0.28 | -0.55 | -0.16 | 0.46 |  |
| ADG x | 0.18 | 0.22 | -0.24 | 0.24 | 0.31 | | 0.29 | -0.02 | -0.19 | 0.16 | 0.40 |  |
| FCR p | 0.66 | 0.39 | 0.38 | 0.08 | 0.21 | | 0.33 | -0.32 | 0.00 | 0.47 | -0.03 |  |
| FCR x | 0.39 | 0.18 | 0.28 | -0.02 | 0.47 | | 0.59 | -0.38 | 0.00 | 0.51 | 0.13 |  |
| ADFI p | 0.45 | 0.49 | 0.00 | 0.36 | 0.71 | | 0.78 | -0.54 | -0.44 | 0.20 | 0.13 |  |
| ADFI x | 0.48 | 0.41 | 0.04 | 0.35 | 0.60 | | 0.68 | -0.46 | -0.37 | 0.39 | 0.28 |  |
| L% p | -0.29 | -0.29 | 0.10 | -0.32 | -0.46 | | -0.53 | 0.56 | 0.54 | -0.26 | -0.01 |  |
| L% x | -0.22 | -0.21 | 0.03 | -0.30 | -0.41 | | -0.48 | 0.42 | 0.40 | -0.37 | -0.28 |  |
| CY p | 0.14 | 0.05 | 0.35 | 0.12 | -0.10 | | 0.05 | 0.18 | -0.12 | -0.15 | 0.04 |  |
| CY x | 0.21 | 0.10 | 0.22 | 0.07 | -0.10 | | 0.04 | -0.10 | -0.40 | -0.49 | -0.40 |  |
| pH Ld p | **0.43** | 0.93 | 0.59 | 0.86 | 0.27 | | 0.19 | -0.64 | -0.84 | -0.20 | -0.45 |  |
| pH Ld x |  | **0.26** | 0.59 | 0.86 | 0.19 | | 0.18 | -0.49 | -0.47 | -0.10 | -0.08 |  |
| pH Ham p | 0.12 | 0.12 | **0.34** | 0.94 | -0.11 | | 0.13 | -0.10 | -0.25 | -0.40 | -0.38 |  |
| pH Ham x | 0.09 | 0.09 |  | **0.28** | -0.31 | | -0.07 | -0.36 | -0.50 | -0.23 | -0.20 |  |
| IF p | 0.15 | 0.25 | 0.21 | 0.25 | **0.44** | | 0.96 | -0.51 | -0.31 | -0.04 | -0.23 |  |
| IF x | 0.20 | 0.18 | 0.21 | 0.25 |  | | **0.44** | -0.34 | -0.13 | 0.32 | 0.20 |  |
| DL p | 0.13 | 0.17 | 0.16 | 0.21 | 0.14 | | 0.19 | **0.62** | 0.97 | 0.08 | 0.20 |  |
| DL x | 0.11 | 0.16 | 0.24 | 0.18 | 0.20 | | 0.20 |  | **0.26** | 0.40 | 0.29 |  |
| ANDR p | 0.14 | 0.24 | 0.16 | 0.23 | 0.17 | | 0.19 | 0.10 | 0.21 | **0.57** | 0.74 |  |
| ANDR x | 0.20 | 0.19 | 0.20 | 0.19 | 0.22 | | 0.15 | 0.19 | 0.12 |  | **0.71** |  |
| TES p | 0.37 | 0.40 | 0.26 | 0.28 | 0.29 | | 0.25 | 0.13 | 0.24 | 0.23 | 0.24 |  |
| TES x | 0.22 | 0.30 | 0.24 | 0.25 | 0.23 | | 0.18 | 0.10 | 0.17 | 0.24 | 0.18 |  |
| OES p | 0.25 | 0.31 | 0.20 | 0.32 | 0.23 | | 0.30 | 0.17 | 0.30 | 0.09 | 0.21 |  |
| OES x | 0.29 | 0.29 | 0.28 | 0.31 | 0.31 | | 0.24 | 0.24 | 0.26 | 0.18 | 0.06 |  |
| LESFE p | 0.21 | 0.28 | 0.17 | 0.21 | 0.26 | | 0.25 | 0.18 | 0.19 | 0.09 | 0.32 |  |
| LESFE x | 0.31 | 0.29 | 0.26 | 0.24 | 0.23 | | 0.22 | 0.21 | 0.20 | 0.10 | 0.17 |  |
| LESBS p | 0.30 | 0.36 | 0.28 | 0.27 | 0.30 | | 0.30 | 0.11 | 0.31 | 0.12 | 0.12 |  |
| LESBS x | 0.30 | 0.26 | 0.25 | 0.27 | 0.19 | | 0.19 | 0.26 | 0.26 | 0.26 | 0.19 |  |
| LESC p | 0.20 | 0.20 | 0.19 | 0.16 | 0.19 | | 0.21 | 0.09 | 0.17 | 0.19 | 0.25 |  |
| LESC x | 0.34 | 0.22 | 0.17 | 0.12 | 0.20 | | 0.20 | 0.11 | 0.17 | 0.25 | 0.17 |  |
| L%CT p | 0.13 | 0.27 | 0.16 | 0.15 | 0.11 | | 0.19 | 0.11 | 0.13 | 0.07 | 0.09 |  |
| L%CT x | 0.18 | 0.28 | 0.18 | 0.17 | 0.14 | | 0.15 | 0.15 | 0.17 | 0.12 | 0.12 |  |
| LEA p | 0.18 | 0.23 | 0.12 | 0.22 | 0.15 | | 0.24 | 0.14 | 0.19 | 0.15 | 0.17 |  |
| LEA x | 0.17 | 0.16 | 0.15 | 0.12 | 0.14 | | 0.14 | 0.15 | 0.18 | 0.17 | 0.12 |  |
| LED p | 0.33 | 0.32 | 0.28 | 0.34 | 0.31 | | 0.31 | 0.26 | 0.43 | 0.36 | 0.46 |  |
| LED x | 0.24 | 0.27 | 0.34 | 0.32 | 0.27 | | 0.15 | 0.26 | 0.33 | 0.19 | 0.14 |  |
| FemD p | 0.22 | 0.33 | 0.24 | 0.30 | 0.17 | | 0.18 | 0.18 | 0.17 | 0.18 | 0.24 |  |
| FemD x | 0.22 | 0.25 | 0.20 | 0.22 | 0.14 | | 0.14 | 0.16 | 0.17 | 0.17 | 0.13 |  |
| HFR p | 0.10 | 0.11 | 0.15 | 0.13 | 0.11 | | 0.15 | 0.13 | 0.16 | 0.09 | 0.13 |  |
| HFR x | 0.11 | 0.11 | 0.13 | 0.09 | 0.13 | | 0.16 | 0.17 | 0.16 | 0.15 | 0.11 |  |

ADG: Average Daily Gain, FCR: Feed Conversion ratio, ADFI: Average Daily Feed Intake, L%: Lean Percentage, CY: Carcass Yield, pH Ld: pH in *Longissimuss Dorsi*, pH Ham: pH in Ham IF: Intramuscular Fat, ANDR: log(Back fat androstenone level), TES: log(plasma testosterone level), OES: log(plasma œstradiol level), LESFE: log(Lesions at Fattening stage Entrance), LESBS: log(Lesions Before Slaughter), LESC: log(Lesions on Carcass), L%CT: Lean percentage with computerized tomography measure, DL: Drip Loss, LEA Loin Eye Area, LED: Loin Eye Density, FemD: Femur Density, HFR: Ham muscle/bone length ratio, SE : standard error.

Table S4 Estimates of heritabilities (diagonal), genetic correlations (above diagonal), and SE estimated (below diagonal) in purebred (p) and crossbred (x) boars with hormones and lesions score.

|  | | Hormones | | | | |  | Lesions score | | | | | | |
| --- | --- | --- | --- | --- | --- | --- | --- | --- | --- | --- | --- | --- | --- | --- |
|  | TES p | | TES x | OES p | OES x | LESFE p | | | LESFE x | LESBS p | LESBS x | LESC p | LESC x |  |
| ADG p | 0.19 | | -0.44 | -0.18 | 0.06 | -0.47 | | | -0.23 | -0.49 | 0.46 | -0.20 | 0.18 |  |
| ADG x | -0.26 | | 0.14 | 0.01 | 0.43 | -0.53 | | | 0.21 | -0.19 | 0.36 | -0.73 | -0.13 |  |
| FCR p | -0.29 | | -0.02 | 0.55 | 0.40 | 0.12 | | | -0.41 | 0.54 | 0.07 | 0.22 | -0.33 |  |
| FCR x | -0.05 | | -0.24 | 0.49 | 0.21 | 0.19 | | | -0.03 | 0.40 | 0.42 | 0.47 | -0.07 |  |
| ADFI p | 0.00 | | -0.27 | 0.34 | 0.34 | -0.55 | | | -0.42 | -0.08 | 0.32 | 0.00 | 0.06 |  |
| ADFI x | 0.26 | | -0.09 | 0.33 | 0.33 | -0.40 | | | 0.13 | 0.21 | 0.54 | -0.31 | -0.16 |  |
| L% p | -0.07 | | 0.03 | -0.18 | -0.01 | 0.42 | | | 0.28 | -0.32 | -0.09 | 0.13 | -0.14 |  |
| L% x | 0.05 | | 0.06 | -0.38 | -0.26 | 0.20 | | | 0.11 | -0.50 | -0.13 | 0.29 | 0.07 |  |
| CY p | -0.01 | | -0.12 | 0.10 | -0.01 | 0.24 | | | 0.21 | -0.16 | 0.41 | 0.20 | -0.04 |  |
| CY x | -0.20 | | -0.05 | -0.46 | -0.56 | -0.03 | | | -0.02 | 0.06 | 0.22 | 0.31 | 0.14 |  |
| pH Ld p | 0.25 | | -0.16 | -0.03 | -0.24 | 0.50 | | | -0.11 | 0.60 | 0.33 | 0.16 | 0.04 |  |
| pH Ld x | 0.39 | | 0.06 | 0.32 | 0.11 | 0.57 | | | 0.08 | 0.34 | 0.45 | 0.62 | 0.52 |  |
| pH Ham p | -0.22 | | -0.47 | -0.48 | -0.37 | 0.26 | | | -0.10 | 0.39 | 0.39 | 0.15 | 0.16 |  |
| pH Ham x | 0.21 | | -0.08 | -0.12 | 0.00 | 0.23 | | | 0.26 | 0.24 | 0.40 | 0.64 | 0.64 |  |
| IF p | 0.00 | | -0.22 | -0.16 | -0.19 | -0.25 | | | -0.10 | -0.21 | 0.51 | -0.01 | -0.43 |  |
| IF x | 0.37 | | 0.13 | 0.07 | 0.04 | 0.11 | | | 0.12 | -0.21 | 0.51 | 0.40 | -0.04 |  |
| DL p | -0.09 | | 0.28 | 0.02 | 0.34 | -0.22 | | | 0.06 | -0.11 | 0.06 | -0.05 | -0.05 |  |
| DL x | 0.05 | | 0.24 | 0.17 | 0.41 | -0.01 | | | 0.11 | -0.22 | -0.23 | 0.15 | 0.16 |  |
| ANDR p | 0.80 | | 0.40 | 0.89 | 0.84 | 0.24 | | | 0.89 | 0.21 | 0.10 | 0.15 | -0.26 |  |
| ANDR x | 0.76 | | 0.27 | 0.80 | 0.96 | -0.03 | | | 0.38 | -0.53 | -0.09 | 0.18 | -0.21 |  |
| TES p | **0.11** | | 0.75 | 0.82 | 0.88 | 0.21 | | | 0.98 | 0.82 | 0.42 | 0.50 | 0.43 |  |
| TES x |  | | **0.29** | 0.57 | 0.67 | 0.67 | | | 0.27 | 0.34 | 0.06 | 0.39 | 0.33 |  |
| OES p | 0.27 | | 0.31 | **0.23** | 0.94 | 0.21 | | | 0.99 | 0.38 | -0.21 | 0.48 | -0.25 |  |
| OES x | 0.23 | | 0.23 |  | **0.18** | 0.04 | | | 0.96 | 0.20 | -0.18 | 0.66 | -0.03 |  |
| LESFE p | 0.32 | | 0.29 | 0.28 | 0.32 | **0.25** | | | 0.20 | 0.74 | 0.17 | 0.74 | 0.84 |  |
| LESFE x | 0.07 | | 0.22 | 0.13 | 0.27 |  | | | **0.25** | -0.30 | 0.24 | 0.23 | 0.05 |  |
| LESBS p | 0.36 | | 0.50 | 0.39 | 0.45 | 0.16 | | | 0.30 | **0.14** | 0.59 | 0.76 | 0.68 |  |
| LESBS x | 0.38 | | 0.32 | 0.27 | 0.27 | 0.38 | | | 0.08 |  | **0.20** | 0.29 | -0.05 |  |
| LESC p | 0.42 | | 0.25 | 0.27 | 0.22 | 0.28 | | | 0.30 | 0.25 | 0.32 | **0.30** | 0.92 |  |
| LESC x | 0.49 | | 0.23 | 0.27 | 0.26 | 0.23 | | | 0.27 | 0.34 | 0.31 |  | **0.36** |  |
| L%CT p | 0.28 | | 0.25 | 0.18 | 0.32 | 0.17 | | | 0.27 | 0.25 | 0.31 | 0.07 | 0.05 |  |
| L%CT x | 0.30 | | 0.24 | 0.16 | 0.35 | 0.32 | | | 0.12 | 0.39 | 0.22 | 0.10 | 0.10 |  |
| LEA p | 0.33 | | 0.24 | 0.21 | 0.24 | 0.22 | | | 0.20 | 0.14 | 0.23 | 0.20 | 0.25 |  |
| LEA x | 0.37 | | 0.13 | 0.27 | 0.21 | 0.38 | | | 0.23 | 0.18 | 0.18 | 0.21 | 0.16 |  |
| LED p | 0.29 | | 0.41 | 0.31 | 0.34 | 0.32 | | | 0.48 | 0.33 | 0.28 | 0.30 | 0.29 |  |
| LED x | 0.51 | | 0.32 | 0.36 | 0.31 | 0.38 | | | 0.38 | 0.10 | 0.30 | 0.21 | 0.24 |  |
| FemD p | 0.33 | | 0.16 | 0.27 | 0.42 | 0.27 | | | 0.30 | 0.36 | 0.35 | 0.24 | 0.28 |  |
| FemD x | 0.30 | | 0.09 | 0.23 | 0.30 | 0.27 | | | 0.09 | 0.40 | 0.17 | 0.20 | 0.22 |  |
| HFR p | 0.24 | | 0.22 | 0.08 | 0.12 | 0.09 | | | 0.20 | 0.26 | 0.21 | 0.18 | 0.24 |  |
| HFR x | 0.29 | | 0.13 | 0.18 | 0.14 | 0.29 | | | 0.16 | 0.38 | 0.16 | 0.23 | 0.17 |  |

ADG: Average Daily Gain, FCR: Feed Conversion ratio, ADFI: Average Daily Feed Intake, L%: Lean Percentage, CY: Carcass Yield, pH Ld: pH in *Longissimuss Dorsi*, pH Ham: pH in Ham IF: Intramuscular Fat, ANDR: log(Back fat androstenone level), TES: log(plasma testosterone level), OES: log(plasma œstradiol level), LESFE: log(Lesions at Fattening stage Entrance), LESBS: log(Lesions Before Slaughter), LESC: log(Lesions on Carcass), L%CT: Lean percentage with computerized tomography measure, DL: Drip Loss, LEA Loin Eye Area, LED: Loin Eye Density, FemD: Femur Density, HFR: Ham muscle/bone length ratio, SE : standard error.

Table S5 Estimates of heritabilities (diagonal), genetic correlations (above diagonal), and SE estimated (below diagonal) in purebred (p) and crossbred (x) boars with CT measurements.

|  | | | | | | CT measurements | | | | | | |
| --- | --- | --- | --- | --- | --- | --- | --- | --- | --- | --- | --- | --- |
|  | L%CT p | L%CT x | LEA p | LEA x | LED p | | LED x | FemD p | FemD x | HFR p | HFR x |  |
| ADG p | -0.16 | -0.72 | -0.09 | -0.62 | 0.17 | | -0.79 | 0.45 | 0.40 | -0.02 | -0.69 |  |
| ADG x | -0.21 | -0.40 | 0.19 | -0.32 | 0.87 | | -0.03 | -0.09 | 0.00 | 0.10 | -0.35 |  |
| FCR p | -0.88 | -0.55 | -0.58 | -0.29 | 0.76 | | 0.31 | 0.44 | 0.13 | -0.72 | -0.35 |  |
| FCR x | -0.88 | -0.68 | -0.56 | -0.26 | 0.63 | | 0.13 | 0.83 | 0.47 | -0.77 | -0.51 |  |
| ADFI p | -0.82 | -0.86 | -0.53 | -0.56 | 0.39 | | -0.27 | 0.72 | 0.39 | -0.58 | -0.76 |  |
| ADFI x | -0.80 | -0.71 | -0.32 | -0.44 | 0.62 | | -0.15 | 0.72 | 0.27 | -0.52 | -0.56 |  |
| L% p | 0.91 | 0.90 | 0.62 | 0.57 | -0.59 | | -0.19 | -0.59 | -0.46 | 0.72 | 0.51 |  |
| L% x | 0.84 | 0.83 | 0.50 | 0.44 | -0.59 | | -0.42 | -0.68 | -0.52 | 0.69 | 0.58 |  |
| CY p | 0.15 | 0.34 | 0.35 | 0.29 | -0.85 | | -0.48 | -0.15 | -0.36 | 0.26 | 0.17 |  |
| CY x | 0.05 | 0.26 | 0.44 | 0.57 | -0.50 | | 0.06 | -0.04 | -0.06 | 0.39 | 0.47 |  |
| pH Ld p | -0.49 | -0.46 | 0.13 | 0.00 | -0.29 | | 0.70 | 0.30 | -0.06 | -0.31 | -0.14 |  |
| pH Ld x | -0.30 | -0.30 | -0.16 | -0.19 | -0.56 | | 0.46 | 0.41 | 0.21 | -0.31 | -0.14 |  |
| pH Ham p | -0.13 | 0.03 | 0.17 | -0.05 | -0.84 | | -0.11 | 0.36 | -0.23 | -0.02 | -0.12 |  |
| pH Ham x | -0.37 | -0.22 | -0.09 | -0.16 | -0.57 | | 0.17 | 0.62 | 0.12 | -0.25 | -0.18 |  |
| IF p | -0.54 | -0.66 | -0.38 | -0.61 | 0.06 | | -0.25 | 0.33 | 0.17 | -0.54 | -0.62 |  |
| IF x | -0.49 | -0.53 | -0.11 | -0.35 | -0.35 | | -0.62 | 0.32 | 0.17 | -0.58 | -0.49 |  |
| DL p | 0.62 | 0.53 | 0.09 | 0.07 | -0.28 | | -0.64 | -0.24 | 0.13 | 0.36 | 0.06 |  |
| DL x | 0.47 | 0.36 | 0.15 | 0.14 | 0.01 | | -0.53 | -0.42 | -0.03 | 0.25 | 0.01 |  |
| ANDR p | -0.37 | -0.47 | -0.31 | -0.50 | 0.39 | | 0.14 | 0.18 | 0.27 | -0.51 | -0.34 |  |
| ANDR x | -0.01 | -0.20 | -0.10 | -0.19 | -0.11 | | -0.05 | -0.34 | 0.13 | -0.10 | -0.26 |  |
| TES p | -0.11 | -0.10 | -0.45 | -0.45 | -0.96 | | -0.42 | -0.30 | 0.26 | -0.09 | -0.21 |  |
| TES x | 0.07 | 0.08 | -0.48 | -0.18 | -0.74 | | 0.05 | -0.63 | 0.03 | -0.19 | 0.15 |  |
| OES p | -0.36 | -0.44 | -0.46 | -0.18 | 0.42 | | -0.24 | 0.00 | -0.16 | -0.38 | -0.12 |  |
| OES x | -0.13 | -0.23 | -0.60 | -0.34 | 0.31 | | -0.34 | 0.02 | 0.09 | -0.32 | -0.50 |  |
| LESFE p | 0.09 | 0.27 | 0.25 | 0.00 | 0.42 | | 0.40 | -0.11 | -0.01 | 0.06 | 0.47 |  |
| LESFE x | 0.38 | 0.06 | 0.11 | -0.08 | -0.38 | | 0.21 | -0.59 | -0.10 | 0.21 | -0.10 |  |
| LESBS p | -0.62 | -0.58 | -0.05 | 0.18 | -0.09 | | 0.89 | 0.41 | 0.02 | -0.24 | -0.17 |  |
| LESBS x | 0.05 | -0.21 | 0.36 | -0.14 | -0.83 | | 0.19 | 0.03 | 0.32 | 0.33 | -0.18 |  |
| LESC p | 0.02 | 0.06 | 0.03 | -0.01 | -0.48 | | -0.69 | 0.08 | -0.02 | -0.10 | -0.21 |  |
| LESC x | 0.03 | 0.08 | 0.19 | 0.09 | -0.12 | | -0.20 | 0.26 | 0.05 | -0.17 | 0.14 |  |
| L%CT p | **0.75** | 1.00 | 0.55 | 0.50 | -0.56 | | -0.09 | -0.79 | -0.48 | 0.79 | 0.63 |  |
| L%CT x |  | **0.71** | 0.60 | 0.63 | -0.63 | | -0.18 | -0.87 | -0.62 | 0.70 | 0.79 |  |
| LEA p | 0.11 | 0.14 | **0.53** | 0.88 | 0.04 | | 0.50 | -0.01 | -0.18 | 0.67 | 0.51 |  |
| LEA x | 0.14 | 0.06 |  | **0.88** | 0.28 | | 0.36 | -0.15 | -0.07 | 0.56 | 0.73 |  |
| LED p | 0.12 | 0.09 | 0.23 | 0.33 | **0.12** | | 0.69 | 0.64 | 0.65 | -0.46 | 0.10 |  |
| LED x | 0.37 | 0.38 | 0.34 | 0.22 |  | | **0.18** | 0.55 | 0.23 | 0.03 | 0.28 |  |
| FemD p | 0.16 | 0.15 | 0.19 | 0.18 | 0.51 | | 0.41 | **0.34** | 0.86 | -0.55 | -0.66 |  |
| FemD x | 0.16 | 0.14 | 0.15 | 0.10 | 0.20 | | 0.33 |  | **0.69** | -0.46 | -0.49 |  |
| HFR p | 0.07 | 0.17 | 0.09 | 0.12 | 0.27 | | 0.32 | 0.20 | 0.20 | **0.67** | 0.82 |  |
| HFR x | 0.16 | 0.12 | 0.22 | 0.06 | 0.27 | | 0.21 | 0.25 | 0.16 |  | **0.76** |  |

ADG: Average Daily Gain, FCR: Feed Conversion ratio, ADFI: Average Daily Feed Intake, L%: Lean Percentage, CY: Carcass Yield, pH Ld: pH in *Longissimuss Dorsi*, pH Ham: pH in Ham IF: Intramuscular Fat, ANDR: log(Back fat androstenone level), TES: log(plasma testosterone level), OES: log(plasma œstradiol level), LESFE: log(Lesions at Fattening stage Entrance), LESBS: log(Lesions Before Slaughter), LESC: log(Lesions on Carcass), L%CT: Lean percentage with computerized tomography measure, DL: Drip Loss, LEA Loin Eye Area, LED: Loin Eye Density, FemD: Femur Density, HFR: Ham muscle/bone length ratio, SE : standard error.
